# Supplementary material for: Impact of FLT3–ITD Mutation Status and Its Ratio in a Cohort of 2901 Patients Undergoing Upfront Intensive Chemotherapy: A PETHEMA Registry Study
Source: Cancers (Basel). 2022 Nov 24;14(23):5799. doi: 10.3390/cancers14235799 (PMC9737662; doi:10.3390/cancers14235799)
Supplement: Supplementary file 1 [file cancers-14-05799-s001.zip › cancers-2005579-supplementary.pdf]

cancers

MDPI

Patients and treatment.

The Spanish PETHEMA LMA Registry (NCT02607059), established in 2013, registers all patients with newly diagnosed acute myeloid leukemia (AML) irrespectively of treatment ("real-world data"). All patients included in this study were treated with intention-to-cure, received intensive chemotherapy based on the combination of cytarabine and an anthracycline, followed or not by allogeneic hematopoietic stem cell transplantation (HSCT) according to predefined risk algorithms.

Patients over 65 years were generally treated according to the PETHEMA LMA 1999 protocol (NCT00464217). Briefly, induction chemotherapy included Ara-C 100 mg/m2/d continuous infusion on days 1 to 7, idarubicin 8 mg/m2/d bolus infusion on days 1 to 3, and GM-CSF (Leucomax) 5 µg/kg subcutaneous or intravenous from day +4 after induction chemotherapy until the recovery of neutropenia (>1.000/mm3). Patients reaching complete remission (CR) after the first cycle received a second cycle to consolidate depth of remission. Patients who do not reached CR after the first cycle received a second cycle after which, in the absence of CR, left the protocol. All patients in CR after one or two cycles of induction received treatment of intensification with Ara-C 500 mg/m2/12h one-hour infusion on days 1 to 4, daunorubicin 45 mg/m2/d bolus infusion on days 5 to 7, and GM-CSF (Leucomax) 5 µg/kg from day +4 after treatment intensification until the recovery of neutropenia (>1.000/mm3).

Patients younger than 65 were generally treated according to the PETHEMA LMA 2007 (NCT01041040) or 2010 (NCT01296178) protocols. In both, induction was based on intravenous idarubicin 12 mg/m2/d on days 1 to 3 plus Ara-C 200 mg/m2/d as a continuous infusion on days 1 to 7. After CR, consolidation consisted of a second identical 3+7 cycle. Patients with high-risk AML (i.e., adverse cytogenetics according to MRC or FLT3-ITD >0.8 and/or ≥0.1% measurable residual disease -MRD- levels by local flow cytometry assessment were recommended to undergo an allogeneic HSCT). Low-risk patients (i.e., CBF leukemia, biallelic mutated CEBPA, normal/intermediate karyotype with mutated NPM1 without FLT3-ITD negative, and/or post-induction MRD <0.1%) were recommended to undergo two additional high-dose cytarabine based consolidation cycles (including or not an autologous HSCT). The remaining patients with intermediate risk were recommended a low-risk post-remission approach unless a matched sibling donor was available; in that case, an allogeneic HSCT in first CR was recommended. The PETHEMA LMA 2007 protocol included administration of single dose Mylotarg during consolidation.

Supplemental Table S1. Characteristics of AML patients cohort study.

Variable	Global Series	Percentage
**Sexo**		
Female	1353	46,70
Male	1544	53,30
**Edad**		
Media (range)	53.6 (12.3-81.2)	
**ECOG**		
0	1016	1016,00
1	1041	1041,00
2	323	323,00
3	69	69,00
4	18	18,00
**WBC count (x1000/mL)**		
Media (SD)	38.2 (0.06-434)	38.2 (0.06-434)
**Cytogenetic Risk**		
Low Risk	238	9,28
Intermediate Risk	1748	68,12
High Risk	580	22,60

**NPM1 mutation**		
Presence	826	31,56
Absence	1791	68,44
**FLT3 ITD mutation**		
Presence	579	19,96
Absence	2322	80,04
**FLT3 ITD ratio levels**		
No mutation	2324	84,72
<0.25	75	2,73
0.25-0.50	82	2,99
0.501-0.80	104	3,79
>0.80	158	5,76
**FLT3 ITD ratio >0.5**		
Presence	273	9,95
Absence	2470	90,05
**FLT3 ITD ratio>0.8**		
Presence	158	5,76
Absence	2585	94,24
**Induction Treatment Squeme**		
Cytarabine and Anthracyclines squeme	2717	93,66
Other combinations	184	6,34
**Induction Treatment Squeme**		
3/7 (Cyt/Ida)	2339	80,63
2/5 (Cyt/Ida)	192	6,62
3/7 (Cyt/Dauno)	72	2,48
FLAG-I	136	4,69
ICE	64	2,21
Ida AraC ajusted to older patient	50	1,72
Mitoxantrone+AraC	48	1,65

Supplemental Table S2. Factors associated to response to induction therapy. UNIVARIATE Regresion Logistic for response to induction treatment.

Variable	OR	Significance	Lower IC	Upper IC
**Sexo**	1.282	p=0.002	1.098	1.495
**Edad**	0.984	p<0.001	0.978	0.989
**WBC (x1000/mL)**	0.998	p<0.001	0.997	0.999
**ECOG**				
0 vs 1	2.622	p=0.044	1.025	6.705
0 vs 2	2.239	p=0.092	0.876	5.721
0 vs 3	1.719	p=NS	0.661	44.469
0 vs 4	1.532	p=NS	0.540	4.351
**Cytogenetic Risk**				
Low Risk vs Intermediate Risk	0.339	p<0.001	0.228	0.503
Low Risk vs Hihg Risk	0.126	p<0.001	0.083	0.190
**NPM1 mutation**				
Absence vs Presence	2.149	p<0.001	1.782	2.591
**FLT3 ITD mutation**				
Absence vs Presence	1.120	p=NS	0.927	1.355
**Ratio FLT3 ITD levels**				
Neg. vs <0.25	1.168	p=NS	0.837	1.631
Neg. vs 0.25-0.50	1.123	p=NS	0.632	1.995
Neg. vs 0.501-0.80	1.731	p=NS	0.958	3.127
Neg. vs >0.80	0.723	p=NS	0.437	1.195
**Ratio FLT3 ITD>0.5**				
Neg. or <0.5 vs >0.5	0.760	p=0.036	0.588	0.982

**Ratio FLT3 ITD>0.8**				
Neg. or <0.8 vs >0.8	0.865	p=NS	0.620	1.206
**Induction Treatment Squeme**				
CYT and anthracyclines combinations vs others combinations	0.747	p=0.060	0.550	1.012
**Induction Treatment Squeme**				
3/7 (I) vs 2/5	0.407	p<0.001	0.303	0.548
3/7 (I) vs 3/7 (D)	0.756	p=NS	0.467	1.224
3/7 (I) vs Flag I	0.828	p=NS	0.578	1.186
3/7 (I) vs ICE	1.444	p=NS	0.815	2.559
3/7 (I) vs 3/7 (older patients)	1.238	p=NS	0.664	2.309
3/7 (I) vs MTZ-ARAC	0.443	p=0.005	0.250	0.785

Supplemental Table S3. Factors associated to death. COX UNIVARIATE for Overall Survival.

Variable	HR	Significance	Lower IC	Upper IC
**Sexo (Male vs Female)**	0.850	p=0.001	0.771	0.937
**Age**	1.030	p<0.001	1.026	1.034
**WBC (x1000/mL)**	1.002	p<0.001	1.001	1.003
**ECOG**				
0 vs 1	1.278	p<0.001	1.138	1.436
0 vs 2	1.710	p<0.001	1.465	1.997
0 vs 3	2.223	p<0.001	1.663	2.971
0 vs 4	3.233	p<0.001	1.935	5.403
**Cytogenetic Risk**				
Low Risk vs Intermediate Risk	1.915	p<0.001	1.532	2.394
Low Risk vs Hihg Risk	3.494	p<0.001	2.762	4.419
**NPM1 mutation**				
Presence vs Absence	0.997	p=NS	0.989	1.005
**FLT3 ITD mutation**				
Presence vs Absence	0.790	p<0.001	0.701	0.890
**Ratio FLT3 ITD levels**				
Neg. vs <0.25	1.142	p=NS	0.827	1.577
Neg. vs 0.25-0.50	1.197	p=NS	0.900	1.592
Neg. vs 0.501-0.80	1.245	p=0.098	0.961	1.612
Neg. vs >0.80	1.547	p<0.001	1.269	1.887
**Ratio FLT3 ITD>0.5**				
Neg. or <0.5 vs >0.5	1.415	p<0.001	1.209	1.657
**Ratio FLT3 ITD>0.8**				
Neg. or <0.8 vs >0.8	1.521	p<0.001	1.248	1.853
**Induction Treatment Squeme**				
3/7 (I) vs 2/5	2.240	p<0.001	1.897	2.645
3/7 (I) vs 3/7 (D)	1.063	p=NS	0.776	1.457
3/7 (I) vs Flag I	1.518	p<0.001	1.233	1.869
3/7 (I) vs ICE	0.948	p=NS	0.702	1.281
3/7 (I) vs 3/7 (older patients)	1.718	p<0.001	1.268	2.329
3/7 (I) vs MTZ-ARAC	2.508	p<0.001	1.855	3.389
**Consolidation (No trasplant; Autotrasplant; Allogeneic trasplant)**				
No Trasp vs Autotrasplant	0.289	p<0.001	0.246	0.338
No Trasp vs Allogeneic Trasplant	0.298	p<0.001	0.258	0.343

Supplemental Table S4. Factors associated to relapse. COX UNIVARIATE for RFS.

Variable	HR	Significance	Lower IC	Upper IC
**Sexo**	1.003	p=NS	0.885	1.136
**Age**	1.012	p<0.001	1.007	1.016
**WBC (x1000/mL)**	1.002	p<0.001	1.001	1.003
**ECOG**				
0 vs 1	1.279	p=0.001	1.101	1.485
0 vs 2	1.520	p<0.001	1.233	1.874
0 vs 3	1.004	p=NS	0.588	1.714
0 vs 4	3.391	p=0.001	1.680	6.843
**Cytogenetic Risk**				
Low Risk vs Intermediate Risk	1.625	p<0.001	1.261	2.095
Low Risk vs Hihg Risk	2.380	p<0.001	1.802	3.143
**NPM1 mutation**				
Absence vs Presence	1.023	p=NS	0.888	1.178
**FLT3 ITD mutation**				
Absence vs Presence	0.804	p=0.006	0.688	0.940
**Ratio FLT3 ITD levels**				
Neg. vs <0.25	0.985	p=NS	0.632	1.535
Neg. vs 0.25-0.50	1.241	p=NS	0.862	1.788
Neg. vs 0.501-0.80	0.911	p=NS	0.625	1.328
Neg. vs >0.80	1.692	p<0.001	1.309	2.188
**Ratio FLT3 ITD>0.5**				
Neg. or <0.5 vs >0.5	1.314	p=0.012	1.062	1.627
**Ratio FLT3 ITD>0.8**				
Neg. or <0.8 vs >0.8	1.686	p<0.001	1.305	2.178
**Induction Treatment Squeme**				
3/7 (I) vs 2/5	1.524	p=0.001	1.183	1.963
3/7 (I) vs 3/7 (D)	0.969	p=NS	0.645	1.454
3/7 (I) vs Flag I	0.951	p=NS	0.692	1.306
3/7 (I) vs ICE	1.050	p=NS	0.737	1.495
3/7 (I) vs 3/7 (older patients)	2.044	p<0.001	1.411	2.961
3/7 (I) vs MTZ-ARAC	1.052	p=NS	0.580	1.908
**Consolidation (No trasplant; Autotrasplant; Allogeneic trasplant)**				
No Trasp vs Autotrasplant	0.564	p<0.001	0.481	0.662
No Trasp vs Allogeneic Trasplant	0.367	p<0.001	0.301	0.424

Supplemental Table SS5. Subgroup analysis of Overall Survival by biological and genomic characteristics.

	**AutoTrasplant** (No. of events/ No.)	**AlloTrasplant** (No. of events/ No.)		HR (95% CI)	
**Sex**					
Male	91/212	127/343	p=NS	1.048 (0.800-1.373)	p=NS
Female	86/217	101/298	p=NS	1.107 (0.830-1.477)	p=NS
**Age**					
<60	129/335	176/520	p=NS	1.173 (0.930-1.480)	p=NS
> or = 60	57/94	52/122	p=0.009	0.988 (0.676-1.444)	p=NS
**Cytogenetic Risk**					
Low	13/69	8/25	p=NS	2.091 (0.858-5.097)	p=0.105
Intermediate	128/301	136/414	p=0.008	0.932 (0.732-1.186)	p=NS
High	18/28	65/140	p=0.084	0.750 (0.441-1.275)	p=NS

**NPM1**					
wild-type	95/223	154/434	p=NS	1.096 (0.850-1.414)	p=NS
mutated	60/165	54/158	p=NS	1.228 (0.848-1.776)	p=NS
**FLT3-ITD**					
wild-type	148/379	173/479	p=NS	1.135 (0.911-1.414)	p=NS
mutated	29/50	55/163	p=0.002	0.767 (0.489-1.203)	p=NS
**FLT3-ratio Group**					
Neg or <0.25	148/380	173/480	p=NS	1.138 (0.913-1.417)	p=NS
0.25-0.50	5/9	4/18	p=0.083	0.656 (0.173-2.488)	p=NS
0.51-0.80	7/11	8/20	p=0.208	0.744 (0.269-2.061)	p=NS
>0.80	2/5	12/38	p=0.706	1.054 (0.233-4.766)	p=NS
**FLT3-ratio >0.5**					
Absence	158/398	184/516	p=NS	1.107 (0.895-1.370)	p=NS
Presence	12/17	34/93	p=0.009	0.665 (0.343-1.288)	p=NS
**FLT3-ratio >0.8**					
Absence	162/405	197/556	p=NS	1.097 (0.891-1.351)	p=NS
Presence	8/10	21/53	p=0.019	0.638 (0.281-1.448)	p=NS
**Overall**	177/429	228/642	p=0.057	1.083 (0.890-1.318)	p=NS

0.12 0.80 1 2 4

FAVORS Allogeneic Trasplant **FAVORS Auto Trasplant**

Supplemental Table SS6. Subgroup analysis of RFS by biological and genomic characteristics.

	**AutoTrasplant** (No. of events/ No.)	**AlloTrasplant** (No. of events/ No.)		HR (95% CI)	
**Sex**					
Male	96/212	92/343	p<0.001	0.689 (0.518-0.918)	p=0.011
Female	110/219	78/300	p<0.001	0.604 (0.452-0.808)	p=0.001
**Age**					
<60	154/337	137/521	p<0.001	0.663 (0.526-0.834) (0.930-1.480)	p<0.001
> or = 60	52/94	33/123	p<0.001	0.601 (0.388-0.930)	p=0.022
**Cytogenetic Risk**					
Low	15/69	11/25	p=0.033	2.411 (1.102-5.273)	p=0.028
Intermediate	149/303	94/415	p<0.001	0.519 (0.401-0.672)	p<0.001
High	20/28	47/141	p<0.001	0.394 (0.231-0.673)	p=0.001
**NPM1**					
wild-type	107/224	115/436	p<0.001	0.650 (0.499-0.846)	p=0.001
mutated	75/166	39/158	p<0.001	0.673 (0.457-0.992)	p=0.046
**FLT3-ITD**					
wild-type	356/563	125/299	p<0.001	0.645(0.512-0.812)	p<0.001
mutated	118/136	45/77	p<0.001	0.515 (0.327-0.810)	p=0.004
**FLT3-ratio Group**					
Neg or <0.25	356/564	126/300	p<0.001	0.652(0.518-0.820)	p<0.001
0.25-0.50	14/16	4/11	P=0.006	0.284 (0.082-0.985)	p=0.047

0.51-0.80	15/19	5/12	p=0.035	0.303 (0.088-1.039)	p=0.058
>0.80	30/32	8/11	p=0.061	0.389 (0.097-1.567)	p=0.184
**FLT3-ratio >0.5**					
Absence	187/400	135/518	p<0.001	0.630 (0.505-0.786)	p<0.001
Presence	11/17	26/93	p=0.003	0.549 (0.270-1.118)	p=NS
**FLT3-ratio >0.8**					
Absence	197/407	143/558	p<0.001	0.622 (0.501-0.772)	p<0.001
Presence	7/10	18/53	p=0.033	0.633 (0.263-1.520)	p=NS
**Overall**	474/699	170/376	p<0.001	0.644 (0.526-0.789)	p<0.001

0.12 0.80 1 2 4

FAVORS Allogeneic Trasplant **FAVORS Auto Trasplant**

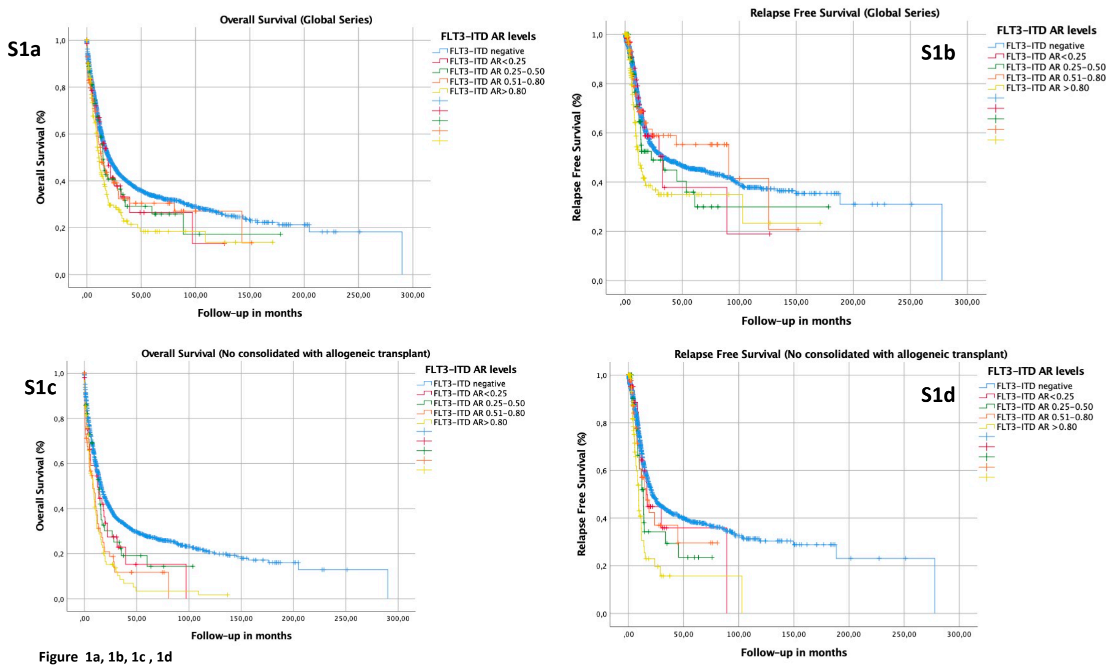

Figure 1a, 1b, 1c , 1d

Supplemental Figure S1. OS (1A) and RFS (1B) in the global series in function of FLT3 ITD AR levels. OS (1A) and RFS (1B) in the no consolidated with allogeneic transplant in function of FLT3 ITD AR levels (**a**). The OS median for FLT3-ITD negative group was 20.4 months (CI 18.1-22.7); FLT3-ITD AR <0.25 was 18.6 months (9.8-27.5); FLT3-ITD AR 0.25-0.50 was 14.8 months (12.8-16.9); FLT3-ITD AR 0.51-0.80 was 13.5 months (7.8-19.3); and FLT3-ITD AR >0.8 was 11.0 months (8.9-13.0) (p<0.001). (**b**) The RFS median for FLT3-ITD negative group was 34.1 months (CI 22.3-45.9); FLT3-ITD AR <0.25 was 32.6 months (13.1-52.2); FLT3-ITD AR 0.25-0.50 was 22.9 months (0-46.3); FLT3-ITD AR 0.51-0.80 was 90.7 months (5.3-176.2); and FLT3-ITD AR >0.8 was 11.8 months (8.5-15.1) (p=0.001). (**c**) The OS median for FLT3-ITD negative group was 15.9months (CI 14.4-17.5); FLT3-ITD AR <0.25 was 12.4months (8.4-16.5); FLT3-ITD AR 0.25-0.50 was 13.9 months (10.1-17.7); FLT3-ITD AR 0.51-0.80 was 8 months (2.2-13.8); and FLT3-ITD AR >0.8 was 8.4 months (5.4-11.5) (p<0.001). (**d**)The RFS median for FLT3-ITD negative group was 21.8 months (CI 18.2-25.4); FLT3-ITD AR <0.25 was 16.8months (10.7-22.9); FLT3-ITD AR 0.25-0.50 was 13.5 months (11.5-15.5); FLT3-ITD AR 0.51-0.80 was 15.9 months (6.6-25.1); and FLT3-ITD AR >0.8 was 9.2 months (8.1-10.2) (p=0.001).

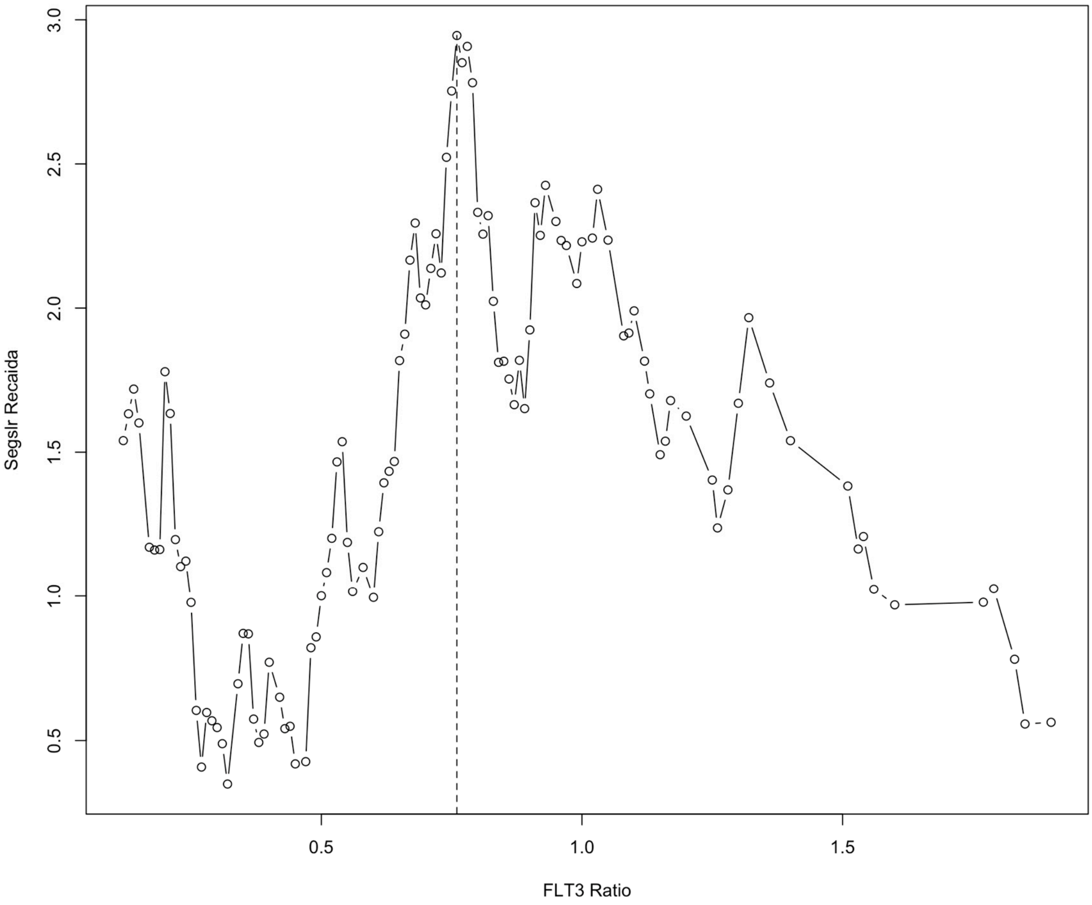

Supplemental Figure S2. Optimal cut-point for the AR by maximally selected log-rank statistics in intensively treated FLT3-ITD positive AMLs for RFS. Maximally selected log-rank statistics performed for the continuum of the AR to test for a potential cut-point separating 2 groups with different survival distributions. The AR is shown on the x-axis and the corresponding standardized log-rank statistic on the y-axis. The estimated cutoff point was 0.77. The vertical dashed line represents the optimal cut-point for AR evident on maximally selected log-rank statistics and the corresponding M statistics. The estimated RFS median in the group with FLT3-ITD AR >0.77 was 9.2months (8.1-10.3) vs 21.3months (18.3-24.3) with lower AR, in the group consolidated with CTX/ AutoHSCT (p<0.001). The estimated RFS median in both groups with FLT3-ITD AR >0.77 and with lower AR, in the group consolidated with allogeneic transplant, were not reached (*p*=NS).

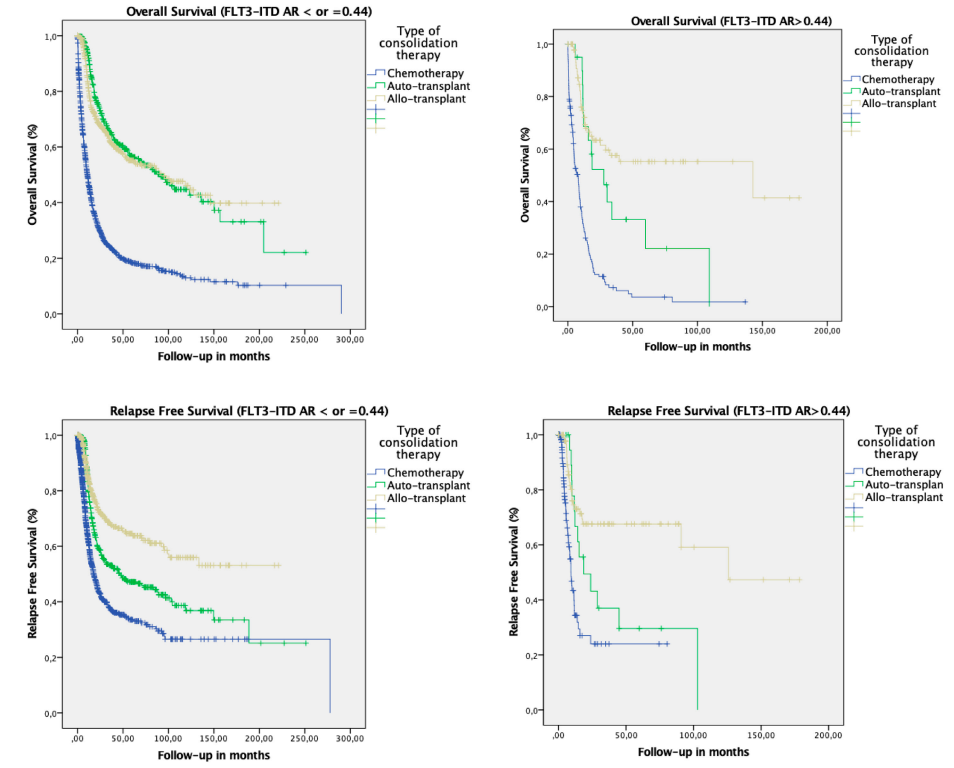

Supplemental Figure S3. (a) The estimated OS median in group with FLT3-ITD AR< or=0.44 was 10.9 (9.8-12.0) for chemotherapy; 91.5 (67.5-115.5) for auto-transplant; 94.5 (53.5-135.4) for allo-transplant: (p<0.001). **(b)** The estimated OS median (CI) in group with FLT3-ITD AR >0.44 was 7.8months (5.1-10.6) for chemotherapy as consolidation therapy; 27.8 (12.8-42.8) for auto-transplant; 142.7 (0-330.46) for allo-transplant (p<0.001). **(c)** The estimated RFS median in group with FLT3-ITD AR< or =0.44 was 17.7 (15.0-20.3) for chemotherapy; 45.9 (18.1-73.7) for auto-transplant; not reached for allo-transplant (p<0.001). **(d)** The estimated RFS median in group with FLT3-ITD AR>0.44 was 9.2 (7.7-10.6) for chemotherapy; 18.7 (2.5-34.9) for auto-transplant; not reached for allo-transplant (p<0.001).

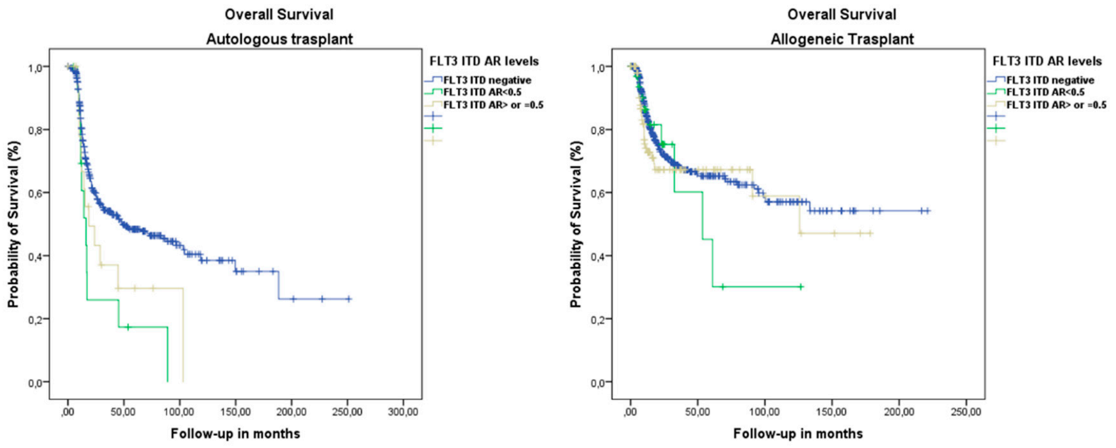

Supplemental Figure S4. Impact on OS according to FLT3-ITD (negative, AR<0,5 or ≥0,5) in AML patients co-occurring with mutated NPM1 in the group consolidated with autologous transplant (**a**) or allogeneic transplant (b). Autologous transplant: The OS median for NPM1-mutated with FLT3-ITD negative group was 48,72 months (CI 18,7-78,71); with FLT3-ITD AR <0.5 was 16,13 months (9,7-22,5); with FLT3-ITD AR ≥0,5 was 18,72 months (2,52-34,9); Allogeneic transplant: The OS

median for NPM1-mutated with FLT3-ITD negative group was not reached; with FLT3-ITD AR <0.5 was 53,51 months (21,37-85,6); with FLT3-ITD AR ≥0,5 was not reached (p=0.013)

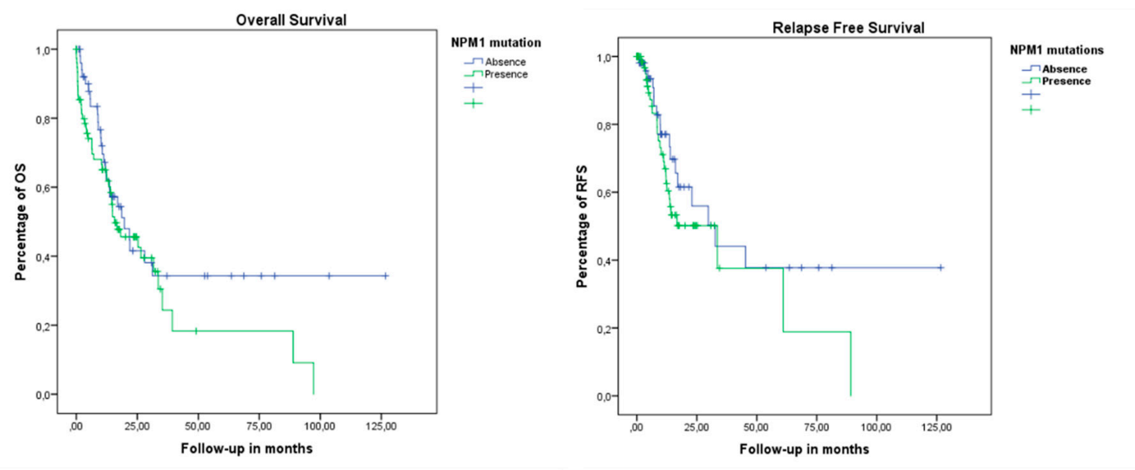

Supplemental Figure S5. Impact on OS and RFS in the group with FLT3 mutated AR <0,5 according the presence or absence of mutated NPM1. (**a**): The OS median for NPM1-wild type was 19,64 months (CI 9,48-29,79) and NPM1-mutated was 15,67 months (5,1-26,27).(**b**). The RFS median for NPM1-wild type was 32,65 months (CI 15,57-49,7) and NPM1-mutated was 33,60 months (7,35-59,8) (p=NS in both comparison).

Appendix

Institutions and clinicians participating in the PETHEMA epidemiologic registry of acute myeloid leukemia and acute promyelocytic leukemia.

Argentina (Grupo Argentino para el Tratamiento de la Leucemia Aguda [GATLA])—Hospital de Clınicas, Buenos Aires: F. Rojas; H. Longoni; Fundaleu, Buenos Aires: G. Milone, I. Fernandez, Clınica Conciencia, Neuquen: R. Ramirez; Hospital Rossi, La Plata: C. Canepa, S. Saba, G. Balladares, Hospital General San Martin, Parana: G. Milone, C. Ventiurini, R. Mariano, P. Negri; Hospital Italiano de La Plata, La Plata: M.V. Prates, J. Milone; Hospital General San Martin, La Plata: P. Fazio, M. Gelemur; Hospital Clemente Alvarez, Rosario: G. Milone, S. Ciarlo, F. Bezares; Hospital de Cordoba, Cordoba: L. Lopez, Hospital Privado de Cordoba, Cordoba: J.J. Garcıa; Instituto Privado Hematologıa, Parana: P. Negri, M. Giunta, G. Milone; Hospital Teodoro Alvarez, Buenos Aires: M. Kruss; Hospital Tornu, Buenos Aires: D. Lafalse, G. Milone; Hospital Gobernador Centeno, La Pampa: E. Marquesoni, M.F. Casale; Hospital Italiano de Buenos Aires, Buenos Aires: A. Gimenez, E.B. Brulc, M.A. Perusini; Complejo Medico Policıa Federal, La Plata: G. Milone, L. Palmer; Colombia (Asociacion Colombian de Hematologıa y Oncologıa [ACHO])—Clınica La Estancia, Popayan: M.E. Correa; Fundacion Valle del Lili, Cauca: F.J. Jaramillo, J. Rosales; Instituto FOSCAL, Bucaramanga: C. Sossa, J.C. Herrera; Hospital Pablo Tobon Uribe, Antioquia: M. Arango; Poland (Polish Adult Leukemia Group [PALG])—City Hospital Legnica, Baja Silesia: J. Holojda; IHIT Hematology and Transfusiology Institute, Warszawa: A. Golos, A. Ejduk; Wojewodzki Szpital Specjalistyczny w Olsztynie, Olsztyn: B. Ochrem; WIM (Military Institute of Medicine in Warsaw),

Warszawa: G. Małgorzata; Poland Medical University of Warsaw Banacha, Warszawa: A. Waszczuk-Gajda, J. Drozd-Sokolowska, M. Czemerska, M. Paluszewska; Medical University School Gdansk, Gdansk: E. Zarzycka; Wojewodzki Szpital Specjalistyczny im. Sw. Jadwigi Sla˛skiej, Opole: A. Masternak; Hospital Brzozow, Brzozow: Dr. Hawrylecka; Medical University Lublin, Lublin: M. Podhoreka, K. Giannopoulos, T. Gromek; Medical University Bialystok, Bialystok: J. Oleksiuk; Silesian Medical University Katowice, Katowice: BA. Armatys, G. Helbig; Universitary Hospital Wroclaw, Wroclaw: M. Sobas; Poznan University of Medical Sciences, Pozna: A. Szczepaniak; Rydigier City

Hospital Krakow, Krakow: E. Rzenno, M. Rodzaj; Collegium Medicum Jagiellonian University Krakow, Krakow: B. Piatkowska-Jakubas; City Hospital Rzeszow, Rzeszow: A. Skret; Medical University Lodz, Lodz: A. Pluta, M. Czemerska; Center of Oncology Kielce, Kielce: E. Baranska; Medical University of Warsaw, Warsaw: M. Paluszewska; Portugal—Hospital de Santa Maria-Lisboa, Lisboa: G. Vasconcelos, J. Brioso; IPOFG Lisboa, Lisboa: A. Nunes, I. Bogalho; Centro Hospitalar e Universitario de Coimbra, Coimbra: A. Espadana, M. Coucelo, S. Marini, J. Azevedo,A.I. Crisostomo, L. Ribeiro, V. Pereira; Centro Hospitalar de Lisboa Central E.P.E., Lisboa: A. Botelho; Instituto Portugues Oncologia do Porto Francisco Gentil, Porto: J.M. Mariz; Centro Hospitalar Sao Joao, Porto: J.E. Guimaraes, E. Aguiar; Centro Hospitalar do Porto E.P.E., Porto: J. Coutinho; Spain (Programa Espanol de Tratamiento de las Hemopatıas Malignas [PETHEMA])—Complejo Hospitalario Universitario A Coruna, A Coruña: V. Noriega, L. Garcıa, C. Varela, G. Deben, M. R. Gonzalez; Hospital Clınico Universitario de Santiago, A Coruna: M. Encinas, A. Bendana, S. Gonzalez, J.L. Bello, M. Albors; Hospital General de Albacete, Albacete: L. Algarra, J.R. Romero, J.S. Bermon, M.J. Varo; Hospital Vinalopo, Alicante: V. Lopez, E. Lopez; Hospital Virgen de los Lirios, Alcoy: C. Mora, C. Amoros; Hospital General Elche, Alicante: E. Lopez, A. Romero; Hospital Torrevieja Salud, Alicante: A. Jaramillo, N. Valdez, I. Molina, A. Fernandez, B. Sanchez; Hospital de la Marina Baixa Villajoyosa, Alicante: A. Garcıa; Hospital General de Elda, Alicante: V. Castano,T. Lopez, J. Bernabeu; Hospital de Denia-Marina Salud, Alicante: M.J. Sanchez; Hospital de la Vega Baja de Orihuela, Alicante: C. Fernandez; Hospital General de Alicante, Alicante: C. Gil, C. Botella, P. Fernandez, M. Pacheco, F. Tarın; J.J. Verdu; Complejo Hospitalario Torrecardenas, Almeria: M.J. Garcıa, A. Mellado, M.C. Garcıa, J. Gonzalez; Hospital Central de Asturias, Asturias: T. Castillo, E. Colado, S. Alonso; Complejo Asistencial Avila, Avila: I. Recio, M. Cabezudo, J. Davila, M.J. Rodrıguez, A. Barez, B. Dıaz; Hospital Don Benito-Villanueva, Badajoz: J. Prieto; Institut Catala d'Oncologia LHospitalet, Barcelona: M. Arnan, C. Martın, M. Mansilla; Hospital de Cruces, Bizkaia: A. Balaberdi, M.E. Amutio, R.A. del Orbe, I. Ancin, J.C. Ruız; Hospital Galdakao-Usansolo, Bizkaia: M. Olivalres, C. Gomez, I. Gonz alez, M. Celis, K. Atutxa, T. Carrascosa, T. Artola, M. Lizuain; Basurtuko Ospitalea, Bizkaia: J.I. Rodriguez, O. Arce, J.A. Marquez, J. Atuch, F. Marco de Lucas, Z. Dıez, B. Davila; Hospital Santos Reyes, Burgos: R. Cantalejo, M. Dıaz; Hospital Universitario de Burgos, Burgos: J. Labrador, F. Serra, G. Hermida, F.J. Dıaz, P. de Vicente, R. Alvarez: Hospital Santiago Apostol, Burgos: C. Alonso, Hospital San Pedro de Alcantara, Caceres: J.M. Bergua; Hospital Campo Aranuelo, Caceres: N. Ugalde; Hospital Virgen del Puerto, Caceres: E. Pardal; Hospital General Jerez de la Frontera, Cadiz: R. Saldana, F. Rodrıguez, E. Martın, L. Hermosın; Hospital Universitario Puerta del Mar, Cadiz: M.P. Garrastazul, I. Marchante, J.A. Raposo, F.J. Capote; Hospital U. Marques de Valdecilla, Cantabria: M. Colorado, A. Batlle, L. Yanez, S. Garcıa, P. Gonzalez, E.M. Ocio, M. Briz, A. Bermudez, S. Garcıa; Consorcio Hospitalario Provincial de Castellon, Castellon: C. Jimenez, S. Beltran; Hospital de Vinaroz: M. Montagud; Hospital Universitario de La Plana, Castellon: I. Castillo; Hospital General de Castellon, Castellon: R. Garcıa, A. Gascon, J. Clavel, A. Lancharro, L. Lnares; Hospital Santa Barbara, Ciudad Real: M.M. Herraez, A. Milena; Hospital Virgen de Altagracia, Ciudad Real: M.J. Romero, Hospital General de Ciudad Real, Ciudad Real: B. Hernandez, C. Calle, R. Benegas; Hospital Gutierrez Ortega de Valdepenas, Ciudad Real: Dr. Bolıvar; Hospital General La Mancha Centro, Ciudad Real: M.A. Pozas; Hospital Reina Sofia, Cordoba: J. Serrano, F.J. Dorado, J. Sanchez, M.C. Martınez; Hospital Virgen de la Luz, Cuenca: C.J. Cervero, M.J. Busto; Hospitales HUVN-HC San Cecilio de Ganada, Granada: M. Bernal, E. Lopez, L. Moratalla, Z. Mesa, M. Jurado, A. Romero, P. Gonzalez; Complejo Hospitalario Universitario Granada, Granada: L. Moratalla, A. Romero, L. Lopez; Hospital Universitario de Guadalajara, Guadalajara: M. Dıaz, D. De Miguel, A.B. Santos, J. Arbeteta; Hospital Donostia, Donosti: E. Perez, N. Caminos, N. Uresandi, N. Argoitiaituart, T. Artola, J. Swen, A. Uranga, I. Olazaba, M. Lizuain, E. Gainza, P. Romero; Hospital Juan Ramon Jimenez Huelva, Huelva: E. Gil, A.J. Palma, K.G. Gomez, M. Sol e, J.N. Rodrıguez; Hospital San Jorge,

Huesca: I.M. Murillo, J. Marco, J. Serena, V. Marco; Hospital de Barbastro, Huesca: M. Perella, L. Costilla; Hospital General Ciudad de Jaen, Jaen: J.A. Lopez, A. Baena, P. Almagro; Hospital San Pedro de Logrono, La Rioja: M. Hermosilla, A. Esteban, B.A. Campeny, M.J. Najera, P. Herrra; Hospital Insular de Las Palmas, Las Palmas: R. Fernandez, J.D. Gonzalez, L. Torres; Hospital Dr. Negrın, Las Palmas: S. Jimenez; M.T. Gomez, C. Bilbao, C. Rodrıguez; Hospital Doctor Jose Molina Orosa, Las Palmas: A. Hong, Y. Ramos de Laon, V. Afonso; Hospital Universitario de Leon, Leon: F. Ramos, M. Fuertes; Hospital Comarcal del Bierzo, Leon: E. de Cabo, C. Aguilera, M. Megido; Hospital Universitari Arnau de Vilanova de Lleida, Leida: T. Garcıa; Hospital Lucus Augusti, Lugo: E. Lavilla, M. Varela, S. Ferrero, M.J. Sanchez, L. Lopez, J. Arias, L. Vizcaya; Hospital Infanta Sofıa, Madrid: A. Roldan, A. Vilches, M.J. Penalva, J. Vazquez; Hospital Central de la Defensa Gomez Ulla, Madrid: M.T. Calderon, A. Matilla, C. Serı, M.J. Otero, N. Garcia, E. Sandoval; Hospital de Fuenlabrada, Madrid: C. Franco, R. Flores, P. Bravo, A. Lopez; Hospital Fundacion Jimenez Dıaz, Madrid: J.L. Lopez, C. Blas, A. Dıez, J.M. Alonso, C. Soto, A. Arenas; Hospital U. Prıncipe de Asturias, Madrid: J. Garcıa, Y. Martın, P.S. Villafuerte, E. Magro; Hospital Puerta de Hierro, Madrid: G. Bautista; A. De Laiglesia; Hospital Gregorio Maranon, Madrid: G. Rodrıguez, L. Solan, M. Chicano, P. Balsalobre, S. Monsalvo, P. Font, D. Carbonell, C. Martınez; Hospital U. La Paz, Madrid: K. Humala, A.E. Kerguelen, D. Hernandez, M. Gasior, P. Gomez, I. Sanchez; Hospital Madrid Norte Sanchinarro, Madrid: S. Redondo, L. Llorente, M. Bengochea, J. Perez; Hospital Sanitas Torrejon, Madrid: A. Sebrango, M. Santero, A. Morales; Hospital La Princesa, Madrid: A. Figuera, P. Villafuerte, A. Alegre, E. Fernandez; Hospital Ruber Internacional, Madrid: A. Alonso; Hospital 12 de Octubre, Madrid: M.P. Martınez, J. Martınez, M. T. Cedena, R. Ayala; MD Anderson Cancer Center, Madrid: A. De la Fuente; Hospital Sanitas La Zarzuela, Madrid: D. Garcıa; Hospital Universitario Quiron, Madrid: C. Chamorro, V. Pradillo, E. Martin, J. M. Sanchez, I. Delgado, A. Alonso; Hospital Rey Juan Carlos, Madrid: B. Rosado, A. Velasco, C. Miranda, G. Salvatierra, J.M. Alonso, J.L. Lopez; Hospital Infanta Leonor, Madrid: M. Foncillas, J.A. Hernandez; Hospital Universitario de Getafe, Madrid: C. Escolano, L. Garcıa, I. Delgado; Hospital Clınico San Carlos, Madrid: C. Benabente, R. Martınez,M. Polo, E. Anguita; Hospital Universitario Severo Ochoa, Madrid: R. Riaza, G. Amores, M.J. Requena; Hospital Universitario Fundacion Alcorcon, Madrid: F. Javier, L. Villalon; Hospital Universitario Moncloa, Madrid: C. Alaez, V. Pradillo, S. Nistal, B. Navas; Hospital Universitario de Mostoles, Madrid: J. Sanchez, M.A. Andreu; Hospital Ramon y Cajal, Madrid: P. Herrera, J. Lopez; Hospital U. Virgen de la Victoria, Malaga: M. Garcıa, M.J. Moreno, A. Fernandez, M.P. Queipo; Hospital Quironsalud Malaga, Malaga: A. Hernandez; Hospital Regional de Malaga, Malaga: M. Barrios, A. Heiniger, A. Jimenez, A. Contento, F. Lopez, M. Alcala; Hospital Vithas Xanit Internacional, Malaga: S. Lorente, M. Gonzalez, E.M. Morales, J. Gutierrez; Hospital Virgen del Castillo, Murcia: M.J. Serna, V. Beltran; Hospital Santa Lucıa de Cartagena, Murcia: M. Romera, M. Berenguer, A. Martınez, A. Tejedor; Hospital Morales Meseguer, Murcia: M. L. Amigo, F. Ortuno, L. Garcıa, A. Jerez, O. Lopez; Hospital U. Virgen de la Arrixaca, Murcia: J.M. Moraleda, P. Rosique, J. Gomez, M.C. Garay; Hospital Los Arcos Mar Menor, Murcia: P. Cerezuela, C. Martınez, A.B. Martınez, A. Gonzalez; Hospital STa Ma del Rosell, Murcia: J. Ibanez; Clınica San Miguel, Navarra: M.J. Alfaro; Complejo Hospitalario de Navarra, Navarra: M. Mateos, M.A. Goni, M.A. Araiz, A. Gorosquieta, M. Zudaire, M. Viguria, A. Zabala, M. Alvarellos, I. Quispe, M.P. Sanchez, G. Hurtado, M. Perez, Y. Burguete, N. Areizaga, T. Galicia; Clınica Universitaria de Navarra, Navarra: J. Rifon, A. Alfonso, F. Prosper, M. Marcos, L.E. Tamariz, V. Riego. A. Manubens, M.J. Larrayoz, M.J. Calasanz, A. Manu, B. Paiva, I. Vazquez, L. Burgos; Complejo Hospitalario de Ourense (CHOU), Ourense: M. Pereiro, M. Rodrıguez, M.C. Pastoriza, J.A. Mendez, J.L. Sastre, M. Iglesias, C. Ulibarrena, F. Campoy; Hospital Valdeorras, Ourense: D. Jaimes; Hospital Rio Carrion, Palencia: J. M. Alonso, B. Albarr an, J. Solano, A. Silvestre; Complexo Hospitalario Universitario de Vigo, Vigo: C. Albo, S. Suarez, C. Loureiro, I. Figueroa, M. Rodrıguez, M.A. Fernandez, A. Martınez, C. Poderos, J. Vazquez, L. Iglesias, A. Nieto, T. Torrado, A.M. Martınez;

Hospital Provincial de Pontevedra, Pontevedra: M.L. Amador, P. Oubina, E. Feijo, A. Dios, I. Loyola, R. Roreno; Hospital POVISA, Pontevedra: A. Simiele, L. Alvarez, V. Turcu; Hospital U. Salamanca, Salamanca: B. Vidriales, M. Gonzalez, R. Garcıa, A. Avendano, C. Chillon, E. Perez, V. Gonzalez; Hospital General La Palma, Santa Cruz de Tenerife: J.V. Govantes, S. Rubio, M. Tapia; Hospital General de Segovia, Segovia: C. Olivier, J.A. Queizan;Hospital U. Virgen Macarena, Sevilla: O. Perez, J.A. Vera, C. Munoz, A. Rodriguez, N. Gonzalez; Hospital U. Virgen del Rocio, Sevilla: J.A. Perez, E. Soria, I. Espigado, J. Falantes, I. Montero, P. Garcıa, E. Rodrıguez, E. Carrillo, T. Caballero, C. Garcıa; Hospital Virgen de Valme, Sevilla: C. Couto, I. Simon, M. G omez; Hospital Virgen del Miron de Soria, Soria: C. Aguilar; Hospital Universitario Canarias, Tenerife: B.J. Gonzalez, S. Lakhwani, A. Bienert, B. Gonzalez; Hospital Universitario Nuestra Senora de Candelaria, Tenrife: A. Cabello, A.Y. Oliva, H. Gonzalez; Hospital Obispo Polanco, Teruel: N. Gonzalez, Hospital de Alcaniz, Teruel: L. Sancho, M. Paricio, L. Perdiguer; Hospital General Nuestra Senora del Prado, Toledo: F. Solano, A. Lerma, M.D. Martınez; Hospital Virgen de la Salud de Toledo, Toledo: M.I. Gomez, A. Yeguas; Hospital U. La Fe, Valencia: P. Montesinos, E. Barragan, C. Sargas, R. Amigo, D. Martinez, B. Boluda, R. Rodrıguez, E. Acuna, I. Cano; Hospital de Requena, Valencia: A. Escriva, M. Pedreno; Hospital de Lluis Alcanyis de Xativa, Valencia: R. Renart; IVO (Instituto Valenciano de Oncologıa), Valencia: A. Navalon; Hospital de Sagunto, Valencia: I. Castillo, M. Orts; Hospital Dr. Peset, Valencia: M.J. Sayas, M.J. Fernandez, M.L. Juan, E. Gomez, M. Gimeno, E. Donato, M. Cejalvo, J. Marco; Hospital Clınico Universitario, Valencia: M. Tormo, M. Calabuig, B. Navarro, I. Martin, E. Villamont, A. Miralles; Hospital de La Ribera, Valencia: R. Lluch; Hospital Casa de la Salud, Valencia: J. Garcıa; Hospital de Gandıa, Valencia: M. Moragues, M.A. Ruiz; Hospital Arnau de Vilanova, Valencia: A. Lopez, C. Benet, M. Valero; Hospital General de Valencia, Valencia: M. Linares, R. Collado, M. Orero, P. Ibanez, M.J. Lis, P.L. Perez, M. Roig, M. Lopez, A.V. Mena; Hospital Manises, Valencia: I. Picon, V. C anovas, A. Palacios, E. Martı; Hospital Clınico de Valladolid, Valladolid: R. Cuello, J. Borrego, M. Burgois; Hospital Rio Hortega, Valladolid: A. Cantalapiedra, O. Norberto, E. Angomas, B. Cidoncha; Hospital Universitario Araba, Victoria: L. Cuevas, D. Robles, A. Mendiazabal, I. Oiartzabal, J.M. Guinea de Castro; Hospital Virgen de la Concha, Zamora: C. Montes, M. Perez, L. Garcıa; Hospital Royo Villanova, Zaragoza: V. Carrasco, A. Perez, L. Lopez, J.J. Moneva; Hospital Clinico U. Lozano Blesa, Zaragoza: M. Olave, E. Bonafonte, L. Mayor, G. Azaceta, L. Palomera; Hospital Ernest Lluch Martin, Zaragoza: M. Malo, M.J. Escobar; Hospital Quiron Salud Zaragoza, Zaragoza: J.M. Grasa; Hospital Miguel Servet, Zaragoza: B. De Rueda, A. Aules, C. Salvador, V. Anso, A. Iborra, P. Delagado, A. Rubio; and Uruguay Hospital de Clınicas, Montevideo: M. Stevenazzi, I. Alpire, V. Irigoin, L. Dıaz, C. Guillermo, R. Guadagna, S. Grille, C. Oliver, M. Boada, V. Vales; Hospital Maciel, Montevideo: A.I. Prado; Cooperativa Medica de Rocha (COMERO), Rocha: A.P. De los Santos.
